# The genome sequence of the chlorophyte *Marvania coccoides* CCAP 251/1B (Naumann) Henley, Hironaka, Guillou, M. Buchheim, J. Buchheim, M. Fawley & K. Fawley 2004

**DOI:** 10.12688/wellcomeopenres.22468.1

**Published:** 2024-06-24

**Authors:** Rachel Allen, Cecilia Rad-Menéndez, David H Green, Christine N. Campbell, Frederik De Boever, Joanne Field, Karen MacKechnie, Michael Ross, Rachel Saxon, Naomi Thomas, Culture Collection of Algae and Protozoa collective, University of Oxford and Wytham Woods Genome Acquisition Lab, Earlham Institute Genome Acquisition Lab and Protists Project, Darwin Tree of Life Barcoding collective, Wellcome Sanger Institute Tree of Life Management, Samples and Laboratory team, Wellcome Sanger Institute Scientific Operations: Sequencing Operations, Wellcome Sanger Institute Tree of Life Core Informatics team, Tree of Life Core Informatics collective, Darwin Tree of Life Consortium

**Affiliations:** 1 Culture Collection of Algae and Protozoa, Oban, Scotland, UK

**Keywords:** Marvania coccoides, green algae, protist, genome sequence, chromosomal, Chlorellales

## Abstract

We present a genome assembly from a culture of *Marvania coccoides* (CCAP 251/1B) (Chlorophyta; Trebouxiophyceae; Chlorellales; Chlorellaceae). The genome sequence is 22.3 megabases in span. Most of the assembly is scaffolded into 13 chromosomal pseudomolecules. The mitochondrial and plastid genome assemblies have lengths of 49.04 kilobases and 99.87 kilobases in length, respectively.

## Species taxonomy

Eukaryota; Viridiplantae; Chlorophyta; core chlorophytes; Trebouxiophyceae; Chlorellales; Chlorellaceae; Marvania (Naumann) Henley, Hironaka, Guillou, M. Buchheim, J. Buchheim, M. Fawley & K. Fawley 2004 (NCBI:txid133487).

## Background

*Marvania coccoides* CCAP 251/1B (Naumann) Henley *et al.*, 2004 is a coccoid freshwater green microalga belonging to the family Chlorellaceae ( Guiry & Guiry, 2011). The Culture Collection of Algae and Protozoa strain (CCAP 251/1B) was isolated by George in 1951 from Cambridge, England (UK). It was initially described as *Nannochloris coccoides,* but its current taxonomic status was established by Henley *et al.* (2004), who reassigned this strain to the *Marvania* genus, and renamed it *Marvania coccoides*. The strain is maintained as a living culture and successfully cryopreserved at CCAP, which ensures long-term safekeeping and open access to viable cultures of this organism.

*M. coccoides* (CCAP 251/1B) has a complicated taxonomic history. *N. coccoides* was one of two species assigned to the *Nannochloris* genus by Naumann (1921). However, neither species was designated as a type at the time and, therefore, the original *N. coccoides* does not have a holotype or any molecular data ( Henley *et al.*, 2004; Temraleeva *et al.*, 2022). In 1981, Hibberd designated *N. coccoides* (CCAP 251/1B) as the lectotype for the genus *Nannochloris* Naumann 1921 as the taxonomic description given by Naumann was a good match for this strain ( Hibberd, 1981). However, Hibberd overlooked the apparent budding of cells during division, which is representative of the species *Marvania geminata* Hindák 1976 ( Tschermak-Woess, 1999), suggesting that the George isolate from 1951 is discrete from the species described by Naumann thirty years earlier ( Henley *et al.*, 2004; Tschermak‐Woess, 1999).

Historically, single-celled coccoid green microalgae have been very challenging to both identify and differentiate, as their morphology is both simple and uniform ( Temraleeva *et al.*, 2022); in the genus *Nannochloris* there is further confusion arising from the high diversity of cell division methods ascribed to it ( Yamamoto *et al.*, 2001). Therefore, molecular analyses are required for identification to a species level ( Temraleeva *et al.*, 2022). Both species described by Naumann were said to reproduce by binary fission, however *N. coccoides* was later found to reproduce by budding and while other species assigned to the *Nannochloris* genus were thought to be polyphyletic, *N. bacillaris* and *N. coccoides* were considered monophyletic ( Yamamoto *et al.*, 2001). This was contradicted by Henley *et al.* (2004) whose molecular analysis of these two species, amongst other related taxa, found that there was a genus-level divergence. On the basis of the budding type of cell reproduction by *N. coccoides* reflecting the similar cell division employed by *M. geminata* (Hindák, 1976), supported by 18s rDNA phylogenetic analysis, it was determined that *Nannochloris coccoides* CCAP 251/1B ought to be reassigned to the *Marvania* genus ( Henley *et al.*, 2004).

## Genome sequence report

**Figure 1.**
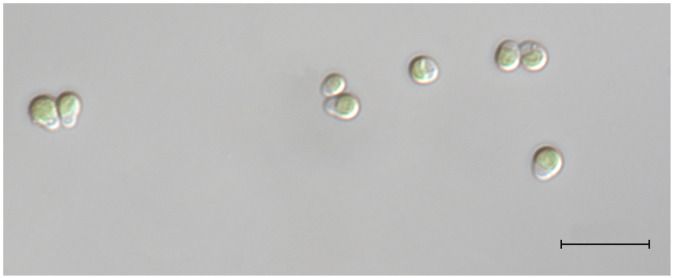
Image of CCAP 251/1B *M. coccoides*. Magnification: x100. Scale bar: 10µm.

The genome was sequenced from *Marvania coccoides* (CCAP 251/1B) cells ( Figure 1) collected from a culture held at the Culture Collection of Algae and Protozoa, Scottish Association for Marine Science. A total of 1,105-fold coverage in Pacific Biosciences single-molecule HiFi long reads was generated. Primary assembly contigs were scaffolded with chromosome conformation Hi-C data. Manual assembly curation corrected one missing join, increasing the assembly length by 0.67% and the scaffold number by 7.14%.

**Figure 2.**
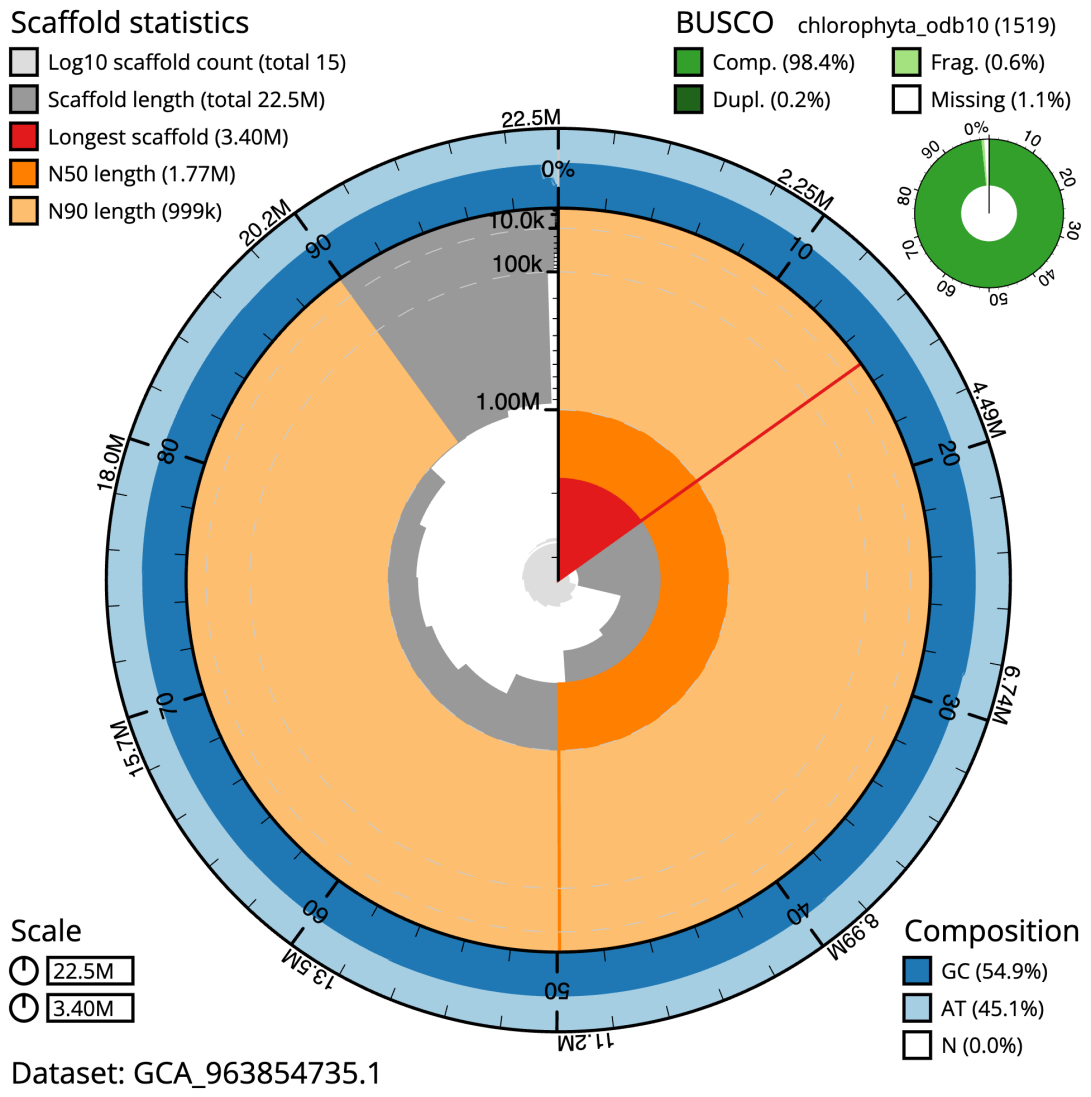
Genome assembly of *Marvania coccoides*, ucMarCocc1.1: metrics. The BlobToolKit snail plot shows N50 metrics and BUSCO gene completeness. The main plot is divided into 1,000 size-ordered bins around the circumference with each bin representing 0.1% of the 22,464,571 bp assembly. The distribution of scaffold lengths is shown in dark grey with the plot radius scaled to the longest scaffold present in the assembly (3,401,833 bp, shown in red). Orange and pale-orange arcs show the N50 and N90 scaffold lengths (1,767,042 and 998,802 bp), respectively. The pale grey spiral shows the cumulative scaffold count on a log scale with white scale lines showing successive orders of magnitude. The blue and pale-blue area around the outside of the plot shows the distribution of GC, AT and N percentages in the same bins as the inner plot. A summary of complete, fragmented, duplicated and missing BUSCO genes in the chlorophyta_odb10 set is shown in the top right. An interactive version of this figure is available at https://blobtoolkit.genomehubs.org/view/Marvania_coccoides/dataset/GCA_963854735.1/snail.

**Figure 3.**
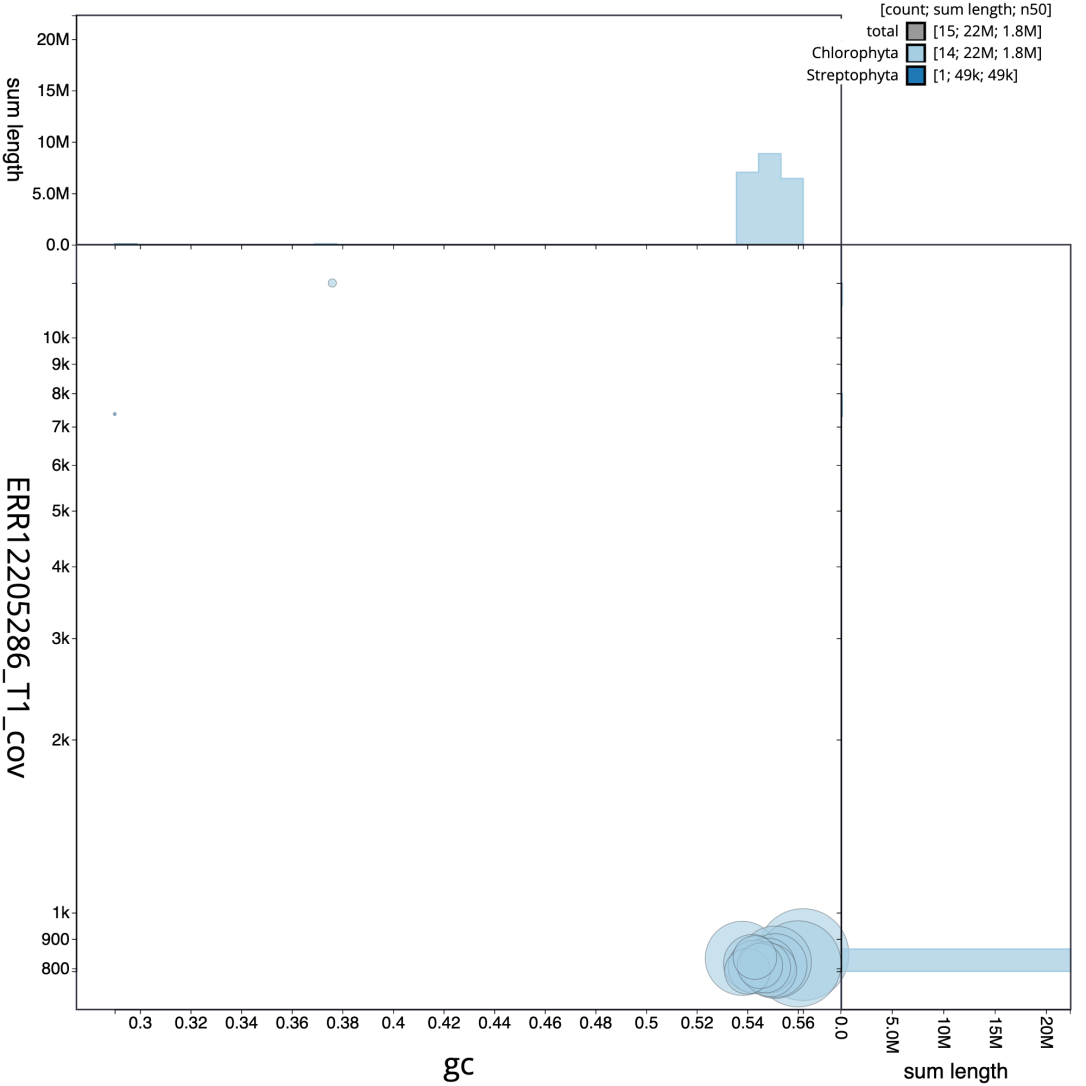
Genome assembly of *Marvania coccoides*, ucMarCocc1.1: BlobToolKit GC-coverage plot. Sequences are coloured by phylum. Circles are sized in proportion to sequence length. Histograms show the distribution of sequence length sum along each axis. An interactive version of this figure is available at https://blobtoolkit.genomehubs.org/view/Marvania_coccoides/dataset/GCA_963854735.1/blob.

**Figure 4.**
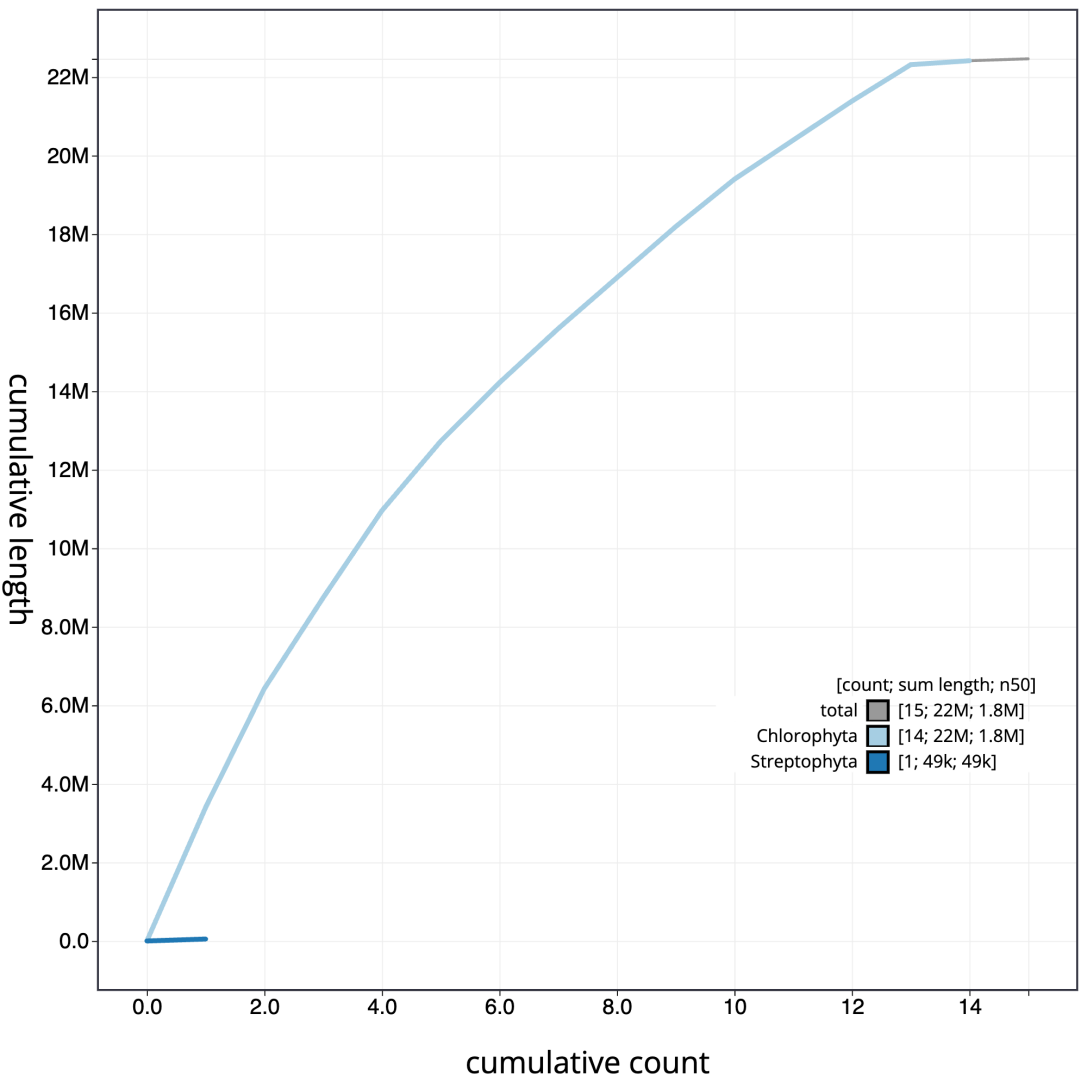
Genome assembly of *Marvania coccoides* ucMarCocc1.1: BlobToolKit cumulative sequence plot. The grey line shows cumulative length for all sequences. Coloured lines show cumulative lengths of sequences assigned to each phylum using the buscogenes taxrule. An interactive version of this figure is available at https://blobtoolkit.genomehubs.org/view/Marvania_coccoides/dataset/GCA_963854735.1/cumulative.

**Figure 5.**
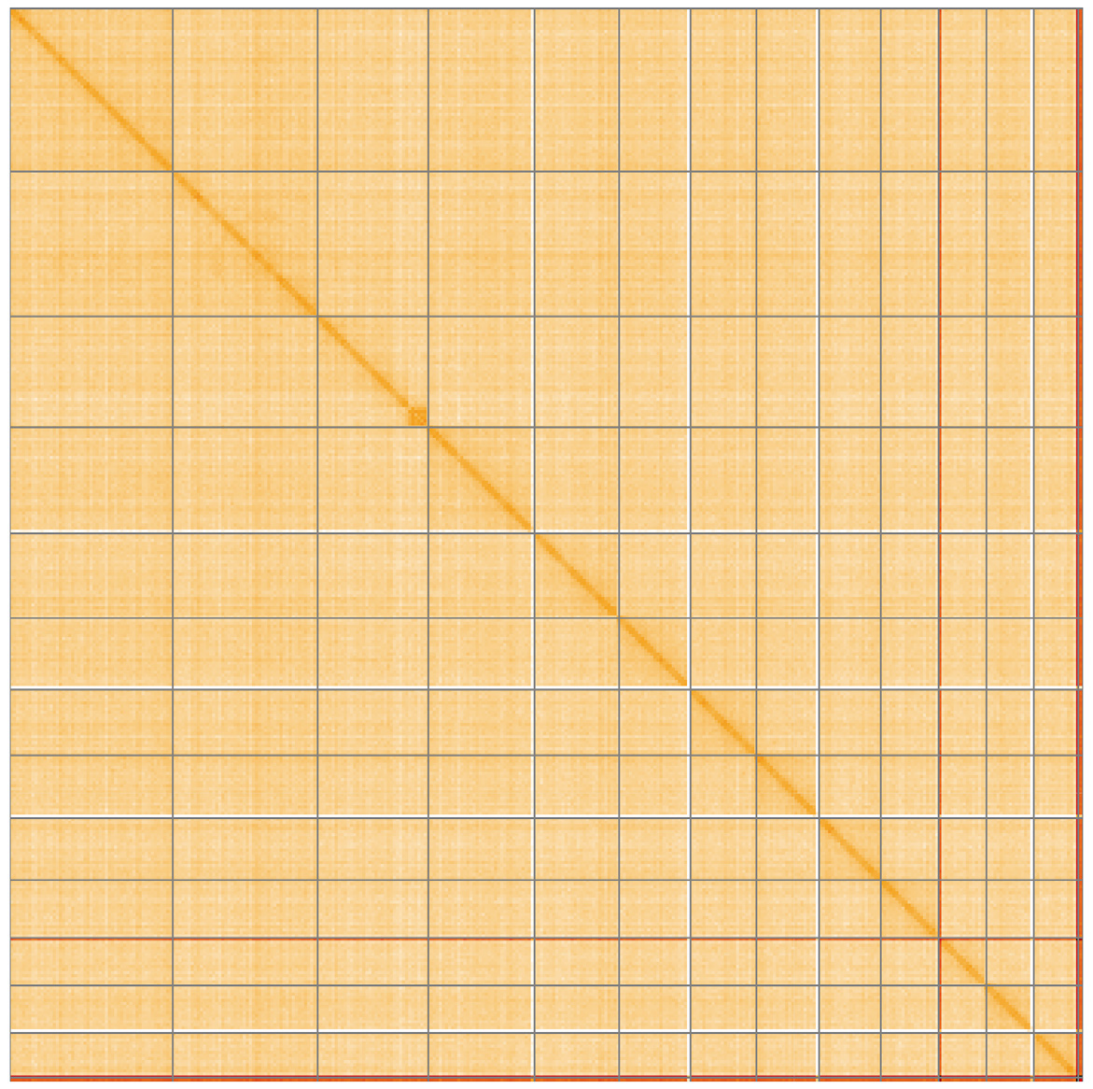
Genome assembly of *Marvania coccoides* ucMarCocc1.1: Hi-C contact map of the ucMarCocc1.1 assembly, visualised using HiGlass. Chromosomes are shown in order of size from left to right and top to bottom. An interactive version of this figure may be viewed at https://genome-note-higlass.tol.sanger.ac.uk/l/?d=IRnji8qfQBaozjid49yULw.

The final assembly has a total length of 22.3 Mb in 13 sequence scaffolds with a scaffold N50 of 1.8 Mb ( Table 1). The snail plot in Figure 2 provides a summary of the assembly statistics, while the distribution of assembly scaffolds on GC proportion and coverage is shown in Figure 3. The cumulative assembly plot in Figure 4 shows curves for subsets of scaffolds assigned to different phyla. Most (99.33%) of the assembly sequence was assigned to 13 chromosomal-level scaffolds. Chromosome-scale scaffolds confirmed by the Hi-C data are named in order of size ( Figure 5; Table 2). While not fully phased, the assembly deposited is of one haplotype. Contigs corresponding to the second haplotype have also been deposited. The mitochondrial and plastid genomes were also assembled and can be found as contigs within the multifasta file of the genome submission.

**Table 1.** Genome data for *Marvania coccoides*, ucMarCocc1.1.

Project accession data		
Assembly identifier	ucMarCocc1.1	
Species	*Marvania coccoides*	
Specimen	ucMarCocc1	
NCBI taxonomy ID	133487	
BioProject	PRJEB68020	
BioSample ID	Genome sequencing: SAMEA12753646 Hi-C scaffolding: SAMEA12753646	
Isolate information	ucMarCocc1, cell culture: (PacBio DNA sequencing and Hi-C sequencing)	
Assembly metrics *		*Benchmark*
Consensus quality (QV)	66.5	*≥ 50*
*k*-mer completeness	100.0%	*≥ 95%*
BUSCO **	C:98.3%[S:98.1%,D:0.2%],F:0.6%,M:1.1%,n:1,519	*C ≥ 95%*
Percentage of assembly mapped to chromosomes	99.33%	*≥ 95%*
Sex chromosomes	None	*localised homologous pairs*
Organelles	Mitochondrial genome: 49.04 kb Plastid genome: 99.87 kb	*complete single alleles*
Raw data accessions		
PacificBiosciences Sequel IIe	ERR12205285, ERR12205286	
Hi-C Illumina	ERR12245615	
Genome assembly		
Assembly accession	GCA_963854735.1	
*Accession of alternate haplotype*	GCA_963694935.1	
Span (Mb)	22.3	
Number of contigs	20	
Contig N50 length (Mb)	1.5	
Number of scaffolds	13	
Scaffold N50 length (Mb)	1.8	
Longest scaffold (Mb)	3.4	

* Assembly metric benchmarks are adapted from column VGP-2020 of “Table 1: Proposed standards and metrics for defining genome assembly quality” from Rhie *et al.* (2021). ** BUSCO scores based on the chlorophyta_odb10 BUSCO set using version 5.4.3. C = complete [S = single copy, D = duplicated], F = fragmented, M = missing, n = number of orthologues in comparison. A full set of BUSCO scores is available at https://blobtoolkit.genomehubs.org/view/Marvania_coccoides/dataset/GCA_963854735.1/busco.

**Table 2.** Chromosomal pseudomolecules in the genome assembly of *Marvania coccoides*, ucMarCocc1.

INSDC accession	Chromosome	Length (Mb)	GC%
OY978097.1	1	3.4	56.0
OY978098.1	2	3.03	56.0
OY978099.1	3	2.31	54.0
OY978100.1	4	2.22	55.0
OY978101.1	5	1.77	55.0
OY978102.1	6	1.49	54.0
OY978103.1	7	1.38	55.0
OY978104.1	8	1.31	54.5
OY978105.1	9	1.29	55.0
OY978106.1	10	1.21	54.5
OY978107.1	11	1.0	54.0
OY978108.1	12	0.99	54.5
OY978109.1	13	0.93	54.5
OY978110.1	MT	0.05	29.0
OY978111.1	Pltd	0.1	37.5

The estimated Quality Value (QV) of the final assembly is 66.5 with *k*-mer completeness of 100.0%, and the assembly has a BUSCO v5.4.3 completeness of 98.3% (single = 98.1%, duplicated = 0.2%), using the chlorophyta_odb10 reference set ( *n* = 1,519).

Metadata for specimens, barcode results, spectra estimates, sequencing runs, contaminants and pre-curation assembly statistics are given at https://links.tol.sanger.ac.uk/species/133487.

## Methods

### Sample acquisition and nucleic acid extraction

*M. coccoides* CCAP 251/1B was cultivated in EG:JM medium ( Jaworski *et al.*, 1981; Schlosser, 1982) at 20°C with ca. 100 µmol m ^-1^ s ^-1^ light for a period of 12:12 h light:dark. Biomass was harvested by centrifugation and snap frozen in liquid nitrogen; samples taken for sequencing (specimen ID Ox800021, ToLID ucMarCocc1) were stored at –80°C and shipped on dry ice.

The workflow for high molecular weight (HMW) DNA extraction at the Wellcome Sanger Institute (WSI) Tree of Life Core Laboratory includes a sequence of core procedures: sample preparation; sample homogenisation, DNA extraction, fragmentation, and clean-up. In sample preparation, the ucMarCocc1 sample was weighed and dissected on dry ice ( Jay *et al.*, 2023). For sample homogenisation, cells were cryogenically disrupted using the Covaris cryoPREP ^®^ Automated Dry Pulverizer ( Narváez-Gómez *et al.*, 2023). HMW DNA was extracted using the Manual MagAttract v1 protocol ( Strickland *et al.*, 2023b). DNA was sheared into an average fragment size of 12–20 kb in a Megaruptor 3 system with speed setting 30 ( Todorovic *et al.*, 2023). Sheared DNA was purified by solid-phase reversible immobilisation ( Strickland *et al.*, 2023a): in brief, the method employs a 1.8X ratio of AMPure PB beads to sample to eliminate shorter fragments and concentrate the DNA. The concentration of the sheared and purified DNA was assessed using a Nanodrop spectrophotometer and Qubit Fluorometer and Qubit dsDNA High Sensitivity Assay kit. Fragment size distribution was evaluated by running the sample on the FemtoPulse system.

Protocols developed by the WSI Tree of Life laboratory are publicly available on protocols.io ( Denton *et al.*, 2023).

### Sequencing

Pacific Biosciences HiFi circular consensus DNA sequencing libraries were constructed according to the manufacturers’ instructions. DNA sequencing was performed by the Scientific Operations core at the WSI on a Pacific Biosciences Sequel IIe instrument. Hi-C data were also generated from ucMarCocc1 using the Arima2 kit and sequenced on the Illumina NovaSeq 6000 instrument.

### Genome assembly and curation

Assembly was carried out with Hifiasm ( Cheng *et al.*, 2021) and haplotypic duplication was identified and removed with purge_dups ( Guan *et al.*, 2020). The assembly was then scaffolded with Hi-C data ( Rao *et al.*, 2014) using YaHS ( Zhou *et al.*, 2023). The assembly was checked for contamination and corrected using the TreeVal pipeline ( Pointon *et al.*, 2023). Manual curation was performed using JBrowse2 ( Diesh *et al.*, 2023), HiGlass ( Kerpedjiev *et al.*, 2018) and PretextView ( Harry, 2022). The mitochondrial and plastid genomes were assembled using MitoHiFi ( Uliano-Silva *et al.*, 2023), which runs MitoFinder ( Allio *et al.*, 2020) or MITOS ( Bernt *et al.*, 2013) and uses these annotations to select the final contigs and to ensure the general quality of the sequences.

### Evaluation of final assembly

The final assembly was post-processed and evaluated with the three Nextflow ( Di Tommaso *et al.*, 2017) DSL2 pipelines “sanger-tol/readmapping” ( Surana *et al.*, 2023a), “sanger-tol/genomenote” ( Surana *et al.*, 2023b), and “sanger-tol/blobtoolkit” ( Muffato *et al.*, 2024). The pipeline sanger-tol/readmapping aligns the Hi-C reads with bwa-mem2 ( Vasimuddin *et al.*, 2019) and combines the alignment files with SAMtools ( Danecek *et al.*, 2021). The sanger-tol/genomenote pipeline transforms the Hi-C alignments into a contact map with BEDTools ( Quinlan & Hall, 2010) and the Cooler tool suite ( Abdennur & Mirny, 2020), which is then visualised with HiGlass ( Kerpedjiev *et al.*, 2018). It also provides statistics about the assembly with the NCBI datasets ( Sayers *et al.*, 2024) report, computes *k*-mer completeness and QV consensus quality values with FastK and MerquryFK, and a completeness assessment with BUSCO ( Manni *et al.*, 2021).

The sanger-tol/blobtoolkit pipeline is a Nextflow port of the previous Snakemake Blobtoolkit pipeline ( Challis *et al.*, 2020). It aligns the PacBio reads with SAMtools and minimap2 ( Li, 2018) and generates coverage tracks for regions of fixed size. In parallel, it queries the GoaT database ( Challis *et al.*, 2023) to identify all matching BUSCO lineages to run BUSCO ( Manni *et al.*, 2021). For the three domain-level BUSCO lineage, the pipeline aligns the BUSCO genes to the Uniprot Reference Proteomes database ( Bateman *et al.*, 2023) with DIAMOND ( Buchfink *et al.*, 2021) blastp. The genome is also split into chunks according to the density of the BUSCO genes from the closest taxonomically lineage, and each chunk is aligned to the Uniprot Reference Proteomes database with DIAMOND blastx. Genome sequences that have no hit are then chunked with seqtk and aligned to the NT database with blastn ( Altschul *et al.*, 1990). All those outputs are combined with the blobtools suite into a blobdir for visualisation.

All three pipelines were developed using the nf-core tooling ( Ewels *et al.*, 2020), use MultiQC ( Ewels *et al.*, 2016), and make extensive use of the Conda package manager, the Bioconda initiative ( Grüning *et al.*, 2018), the Biocontainers infrastructure ( da Veiga Leprevost *et al.*, 2017), and the Docker ( Merkel, 2014) and Singularity ( Kurtzer *et al.*, 2017) containerisation solutions.

Table 3 contains a list of relevant software tool versions and sources.

**Table 3.** Software tools: versions and sources.

Software tool	Version	Source
BEDTools	2.30.0	https://github.com/arq5x/bedtools2
Blast	2.14.0	ftp://ftp.ncbi.nlm.nih.gov/blast/executables/blast+/
BlobToolKit	4.3.7	https://github.com/blobtoolkit/blobtoolkit
BUSCO	5.4.3 and 5.5.0	https://gitlab.com/ezlab/busco
bwa-mem2	2.2.1	https://github.com/bwa-mem2/bwa-mem2
Cooler	0.8.11	https://github.com/open2c/cooler
DIAMOND	2.1.8	https://github.com/bbuchfink/diamond
fasta_windows	0.2.4	https://github.com/tolkit/fasta_windows
FastK	427104ea91c78c3b8b8b49f1a7d6bbeaa869ba1c	https://github.com/thegenemyers/FASTK
GoaT CLI	0.2.5	https://github.com/genomehubs/goat-cli
Hifiasm	0.16.1-r375	https://github.com/chhylp123/hifiasm
HiGlass	44086069ee7d4d3f6f3f0012569789ec138f42b84a a44357826c0b6753eb28de	https://github.com/higlass/higlass
MerquryFK	d00d98157618f4e8d1a9190026b19b471055b22e	https://github.com/thegenemyers/MERQURY.FK
MitoHiFi	2	https://github.com/marcelauliano/MitoHiFi
MultiQC	1.14, 1.17, and 1.18	https://github.com/MultiQC/MultiQC
NCBI Datasets	15.12.0	https://github.com/ncbi/datasets
Nextflow	23.04.0-5857	https://github.com/nextflow-io/nextflow
PretextView	0.2	https://github.com/wtsi-hpag/PretextView
purge_dups	1.2.3	https://github.com/dfguan/purge_dups
samtools	1.16.1, 1.17, and 1.18	https://github.com/samtools/samtools
sanger-tol/ genomenote	1.1.1	https://github.com/sanger-tol/genomenote
sanger-tol/ readmapping	1.2.1	https://github.com/sanger-tol/readmapping
Seqtk	1.3	https://github.com/lh3/seqtk
Singularity	3.9.0	https://github.com/sylabs/singularity
TreeVal	1.0.0	https://github.com/sanger-tol/treeval
YaHS	1.1a.2	https://github.com/c-zhou/yahs

### Wellcome Sanger Institute – Legal and Governance

The materials that have contributed to this genome note have been supplied by a Darwin Tree of Life Partner. The submission of materials by a Darwin Tree of Life Partner is subject to the **‘Darwin Tree of Life Project Sampling Code of Practice’**, which can be found in full on the Darwin Tree of Life website here. By agreeing with and signing up to the Sampling Code of Practice, the Darwin Tree of Life Partner agrees they will meet the legal and ethical requirements and standards set out within this document in respect of all samples acquired for, and supplied to, the Darwin Tree of Life Project.

Further, the Wellcome Sanger Institute employs a process whereby due diligence is carried out proportionate to the nature of the materials themselves, and the circumstances under which they have been/are to be collected and provided for use. The purpose of this is to address and mitigate any potential legal and/or ethical implications of receipt and use of the materials as part of the research project, and to ensure that in doing so we align with best practice wherever possible. The overarching areas of consideration are:

• Ethical review of provenance and sourcing of the material

• Legality of collection, transfer and use (national and international)

Each transfer of samples is further undertaken according to a Research Collaboration Agreement or Material Transfer Agreement entered into by the Darwin Tree of Life Partner, Genome Research Limited (operating as the Wellcome Sanger Institute), and in some circumstances other Darwin Tree of Life collaborators.

## Data Availability

European Nucleotide Archive: *Marvania coccoides*. Accession number PRJEB68020; https://identifiers.org/ena.embl/PRJEB68020 ( Wellcome Sanger Institute, 2023). The genome sequence is released openly for reuse. The *Marvania coccoides* genome sequencing initiative is part of the Darwin Tree of Life (DToL) project. All raw sequence data and the assembly have been deposited in INSDC databases. The genome will be annotated using available RNA-Seq data and presented through the Ensembl pipeline at the European Bioinformatics Institute. Raw data and assembly accession identifiers are reported in Table 1.

## Author information

Members of the Earlham Institute Genome Acquisition Lab and Protists Project are given here: https://doi.org/10.5281/zenodo.8395242.

Members of the Culture Collection of Algae & Protozoa are given here: https://doi.org/10.5281/zenodo.7650573.

Members of the University of Oxford and Wytham Woods Genome Acquisition Lab are listed here: https://doi.org/10.5281/zenodo.7125292.

Members of the Darwin Tree of Life Barcoding collective are listed here: https://doi.org/10.5281/zenodo.4893703.

Members of the Wellcome Sanger Institute Tree of Life Management, Samples and Laboratory team are listed here: https://doi.org/10.5281/zenodo.10066175.

Members of Wellcome Sanger Institute Scientific Operations: Sequencing Operations are listed here: https://doi.org/10.5281/zenodo.10043364.

Members of the Wellcome Sanger Institute Tree of Life Core Informatics team are listed here: https://doi.org/10.5281/zenodo.10066637.

Members of the Tree of Life Core Informatics collective are listed here: https://doi.org/10.5281/zenodo.5013541.

Members of the Darwin Tree of Life Consortium are listed here: https://doi.org/10.5281/zenodo.4783558.
