# Supplementary material for: The genome sequence of the chlorophyte Marvania coccoides CCAP 251/1B (Naumann) Henley, Hironaka, Guillou, M. Buchheim, J. Buchheim, M. Fawley & K. Fawley 2004
Source: Wellcome Open Res. 2024 Jun 24;9:334. [Version 1] doi: 10.12688/wellcomeopenres.22468.1 (PMC11833223; doi:10.12688/wellcomeopenres.22468.1)
Supplement: Peer review history

# Reviewer report: Reviewer response for version 1

**DOI:** 10.21956/wellcomeopenres.24756.r118096

**Published:** 2025-02-17

**Authors:** Shigekatsu Suzuki

**Affiliations:** 1 National Institute for Environmental Studies, Tsukuba, Japan

Allen et al. report the high-quality genome of a unicellular trebouxiophycean *Marvania coccoides*. This genomic information can contribute to understanding diversity of trebouxiopnyceans. I have some minor concerns about this manuscript and propose indexing after revision.

 3 ^rd^ paragraph of Background. The 3rd paragraph has many duplications, and it is difficult to understand. Please make it clearer. I propose that you should only describe the difference in the cell division mode and phylogenetic conclusions here.

 2 ^nd^ paragraph of Genome sequence report. If you know the putative chromosome number, please describe it here. Are there telomere-like sequences at both ends of the pseudochromosomes?

 1 ^st^ paragraph of Methods. Is this strain axenic? If so, an analysis using blobtools (fig. 3) is not necessary. This analysis might be misleading of any contamination.

Are sufficient details of methods and materials provided to allow replication by others?

Yes

Is the rationale for creating the dataset(s) clearly described?

Yes

Are the datasets clearly presented in a useable and accessible format?

Yes

Are the protocols appropriate and is the work technically sound?

Yes

Reviewer Expertise:

phycology, genomics

I confirm that I have read this submission and believe that I have an appropriate level of expertise to confirm that it is of an acceptable scientific standard.

# Reviewer report: Reviewer response for version 1

**DOI:** 10.21956/wellcomeopenres.24756.r101592

**Published:** 2024-10-02

**Authors:** David Nelson

**Affiliations:** 1 New York University Abu Dhabi, Abu Dhabi, United Arab Emirates

The authors describe the sequencing and assembly of the chlorophyte Marvania coccoides. The work is timely and well-prepared. The Nannochloris/Marvania genera are extremely interesting organisms and are great foci for genomics work. The assembly looks good. As this is a 'Data Note' format, I didn't expect any comparative analyses, so I think the amount of information is fine.

 The underlying data is already publicly available and will serve as a great asset to the community. I couldn't detect any obvious flaws with the work. Recommend for Indexing.

Are sufficient details of methods and materials provided to allow replication by others?

Yes

Is the rationale for creating the dataset(s) clearly described?

Yes

Are the datasets clearly presented in a useable and accessible format?

Yes

Are the protocols appropriate and is the work technically sound?

Yes

Reviewer Expertise:

Microalgal genomics. I've sequenced and assembled similar species' genomes. This one looks good.

I confirm that I have read this submission and believe that I have an appropriate level of expertise to confirm that it is of an acceptable scientific standard.
